# Fake Science: XMRV, COVID-19, and the Toxic Legacy of Dr. Judy Mikovits

**DOI:** 10.1089/aid.2020.0095

**Published:** 2020-07-02

**Authors:** Stuart J.D. Neil, Edward M. Campbell

**Affiliations:** ^1^ Department of Infectious Disease, School of Immunobiology and Microbial Sciences, King's College London, London, United Kingdom.; ^2^ Department of Microbiology and Immunology, Stritch School of Medicine, Loyola University Chicago, Chicago, Illinois, USA.

**Keywords:** XMRV, endogenous retroviruses, epidemiology, SARS-CoV2, COVID-19

## Abstract

One cannot spend >5 min on social media at the moment without finding a link to some conspiracy theory or other regarding the origin of SARS-CoV2, the coronavirus responsible for the COVID-19 pandemic. From the virus being deliberately released as a bioweapon to pharmaceutical companies blocking the trials of natural remedies to boost their dangerous drugs and vaccines, the Internet is rife with far-fetched rumors. And predictably, now that the first immunization trials have started, the antivaccine lobby has latched on to most of them. In the last week, the trailer for a new “bombshell documentary” *Plandemic* has been doing the rounds, gaining notoriety for being repeatedly removed from YouTube and Facebook. We usually would not pay much heed to such things, but for retrovirologists like us, the name associated with these claims is unfortunately too familiar: Dr. Judy Mikovits.

May is the month when we and colleagues in our close-knit family of retrovirus researchers usually descend on the Cold Spring Harbor Laboratories on Long Island for a week of camaraderie and intense scientific discussion. It was at this meeting and others that the full scope of Judy Mikovits fraud and malfeasance became clear to the research community, so clear that we came to expect, if not hope that we would never have to hear her name again. Yet here she was popping up all over the right-wing news, reeling off a story of how COVID-19 was the result of animal experimentation in the development of vaccines, and how vaccines themselves were contaminated with mouse retroviruses that were causing untold ills. Naturally this information, and her research in particular, was being suppressed by Big Pharma and the “corrupt mainstream scientists” on their payroll. And the villain of the piece is none other than Dr. Anthony Fauci, the head of the National Institute of Allergy and Infectious Disease and long-suffering clinical advisor to Trump's COVID-19 Taskforce. The trouble with this conspiracy theory is that not only is it demonstrably untrue, much of it derives from a scientific fraud that Mikovits and coworkers perpetrated in 2009.

So, to understand how this took place, we need to give the uninitiated reader a little bit of background on retroviruses. They are an ancient family of viruses whose genetic material is not DNA, but a related molecule, RNA. When a retrovirus infects an animal cell, the viral RNA is converted into DNA and then inserted directly into the genome of the cell.^1^ This means that as long as that cell lives, the viral genome will persist in the infected cell. It will also be passed on to daughter cells should that infected cell divide. Acting essentially like a cellular gene, the virus then makes copies of its RNA genome and packages them into new virus particles that go on to infect the next cell. A curious feature of retroviruses results from their lifestyle. On rare occasions, a retrovirus will infect a germ cell, that is, one that becomes either an egg or a sperm cell. That means that when that cell forms a new organism, the retrovirus will be a part of the genome of every cell in the body and all generations from then on.^2^ The genomes of mammals have become littered with a fossil record of retrovirus infection, most of which is now inactive. Around 8% of the human genome is made of dead retroviruses and the study of these “endogenous retroviruses” provides fascinating insight into our evolution. However, some of these endogenous retroviruses are still able to be reactivated, and mice, in particular, harbor many that can be “reawoken.”

Until 2006, there were four retroviruses known to cause human disease. Then an article was published by Bob Silverman and coworkers in Ohio and Seattle. They had been using a new technology to identify novel viruses in prostate cancer, and they had got a hit. In a couple of biopsies, and crucially in a long-established human prostate cancer cell line known as 22RV1, they identified a new retrovirus that was highly similar to mouse viruses that cause leukemia.^3^ They gave this virus the rather ungainly name xenotropic murine-leukemia virus-related virus (XMRV). Although there was some concern about these data, XMRV had some attributes that made it attractive. It was “xenotropic” meaning that it could infect cells of mammals other than mice, including those from humans. It was part of the family of “simple retroviruses” known to cause cancer in mice. And finally, promoter sequences at the left-hand end of the viral genome that are important for directing the production of new viral RNA genomes in infected cells contained elements that would be specifically activated by male hormones—perfect for viral replication in the prostate.^4,5^ In fact, these promoter sequences proved to be problematic experimentally, as the virus did not replicate well in other cultured cell lines. So much so in fact that when the Silverman laboratory cloned the virus as a DNA form, they replaced these sequences with those that allowed XMRV to replicate in any cell type—sequences known as a cytomegalovirus (CMV) promoter.^6^ This key facet will become important in what follows later.

Judy Mikovits had worked on human retroviruses at the National Cancer Institute throughout the late 1980s and early 1990s with her mentor, Frank Ruscetti.^7^ She had published steadily, if unremarkably, mainly on the activity of the aforementioned promoter sequences of the retroviruses human T cell leukemia virus and HIV-1. After changing fields and moving into the private sector, Mikovits became the scientific director of a small private research institute, the Whittemore Peterson Institute (WPI), in Reno, Nevada.^7^ The WPI had been set up by a local millionaire businessman and his wife to investigate the causes of “neuroimmune diseases” and particularly myalgic encephalomyelitis (ME), also known as chronic fatigue syndrome (CFS), that had stricken their daughter. ME/CFS was a controversial area (reviewed in Ref.^8^). It lacked a robust case definition, its symptoms varied widely but could be incredibly debilitating, often associated with mental health problems. And although similar symptoms can be seen in people recovering from a number of serious infections, ME/CFS sufferers complained of not being taken seriously and being accused of malingering. Mainstream medical opinion was divided, but psychiatrists had found that some ME/CFS sufferers benefitted from cognitive behavioral therapy. These findings were hugely controversial with ME/CFS activists resistant to their (erroneous) interpretation that the disease was “all in their head.” On the extremes of the ME/CFS community, some were asking whether there was an unknown agent causing their suffering that was being covered up.

The story broke in 2009 in a now infamous article authored by Mikovits's team and published in *Science*.^9^ In it they claimed to have found stunning evidence of XMRV in the majority blood samples of people with ME/CFS. Upon finding these initial results, Mikovits apparently contacted the Silverman group in Ohio to ask for some of their XMRV DNA to help them confirm their findings. Mikovits also then sent the WPI ME/CFS and control samples to Silverman to reproduce the detection of XMRV independently. Even more surprising data followed. The purified white blood cells of the ME/CFS sufferers were chock-full of XMRV proteins. Finally, not only could Mikovits detect the XMRV genome in the blood of ME/CFS samples, but a minority of healthy control bloods also appeared to have detectable virus. If true, the implications of this study were huge. First, ME/CFS was potentially caused by a novel human retrovirus, opening up whole new avenues for studying and treating the condition, while also validating the campaigners' notion that it was a “real” illness. Second, picking up 2 positives in 50 healthy controls, if extrapolated to the U.S. population, meant millions of people were harboring an undiagnosed retrovirus infection that now was associated with prostate cancer and ME/CFS. What else might XMRV cause? And importantly, given the sad history of HIV/AIDS and hepatitis C, concern was raised that XMRV had contaminated blood banks.

Understandably, therefore, Mikovits's XMRV article generated a lot interest. However, many retrovirologists had been here before. XMRV was highly similar to endogenous retroviruses found in mice and other mammals, and with the amount of mouse DNA kicking around many laboratories, the sensitive tests that detected the presence of XMRV DNA in samples were prone to contamination.^10–12^ It would not be the first time that a novel human retrovirus turned out to be a laboratory contamination,^13^ and this needed to be ruled out by independent studies as soon as possible. Moreover, from a scientific point of view, the prospect of a new human retrovirus to study was very exciting, and many groups decided to do some pilot experiments to understand the biology of XMRV and whether it really was associated with ME/CFS or prostate cancer.

The attention brought to XMRV led to flurry of studies trying to confirm whether the virus could be detected in ME/CFS blood sample. To do this is very easy: one orders a set of fairly cheap reagents from a company and within 2–3 days you have the tools to test samples. Any decent molecular biology laboratory can do this. Laboratories struggled with precisely the same issues now discussed on the news regarding SARS-CoV2 testing, with the key being to demonstrate that the test is sensitive enough to detect very low number of copies of the virus while avoiding false positives. Once that is done, you are away, and the wheels started to come off of the XMRV bandwagon almost immediately.

First out of the blocks was a collaborative effort between virologists at Imperial College London and Professor Sir Simon Wessely from King's College London who had been studying ME/CFS for some time and had led studies that showed the benefit of behavioral therapy in managing patients.^14^ Not a single stored sample, ME/CFS or otherwise, came up positive. The study was rapidly published, and there was an immediate backlash. Mikovits claimed that the study was not a true replication study and was, therefore, invalid. A group of obviously unrepresentative, but highly militant, ME/CFS activists began to blanket the Internet with the usual conspiracy—the study was a put-up job backed by sinister forces who did not want the world to know that XMRV was literally killing swathes of the population. This was accompanied by what became a very common refrain—“In Judy We Trust.” The lead virologist at Imperial, Prof. Myra McClure, even received death threats. Further U.K. studies were stymied on the eve of publication.^15^ ME/CFS sufferers who had originally consented to taking part were encouraged by the same activists to withdraw it *en masse* when it became known that they too could find no evidence of XMRV in the blood samples.^16^ Other studies elsewhere in the world also failed to corroborate Mikovits's findings. She and her colleague Vincent Lombardi posted rebuttals on the WPI website every time a new one came out. The WPI had already offered diagnostic tests to ME/CFS sufferers to tell them their “XMRV status,” further fanning the flames. How could they be wrong if individuals with ME/CFS were being told they were “XMRV positive”? Then an article from Harvey Alter, a clinician who first recognized hepatitis C, found evidence of murine leukemia virus-like sequences in both CFS and healthy blood samples.^17^ And these authors claimed to have identified the human DNA either side of the integrated retrovirus—good evidence of infectious XMRV in the sample. But there was an issue. To detect the signal, Alter's colleagues had amplified the viral DNA vastly more than is normal in a diagnostic test, greatly increasing the risk of picking up a contaminant. Despite this, Mikovits hailed the article as a validation of her study.

There was something strange about the Alter study, first noticed by Stephane Hue in Prof. Greg Towers' group at University College London.^18^ They had started looking at the 22RV1 prostate cancer cell line that Bob Silverman had shown was persistently infected and producing live XMRV. 22RV1 contains multiple copies of XMRV integrated in its genome.^19^ Critically, when retroviruses copy their RNA genome into DNA for integration, they are very error prone. They make mistakes, or mutations, in the sequence. In fact, they make these mistakes so regularly, that in an individual infected with a retrovirus like HIV-1, there is no one virus sequence, but rather a group or “swarm” of related sequences. This is a hallmark of retrovirus replication—so much so that a lack of sequence diversity means the virus is not actually replicating. What Hue realized was that there were more mutations between the different XMRV integrations in the 22RV1 cell line than in the sequences fished out of CFS samples by Alter. In fact, these sequences were identical to one of the 22RV1 viruses. This was very compelling evidence that not only could not XMRV be replicating in these people, it was very likely to be a laboratory contamination from the 22RV1 cell line. In their excitement to publish this, they at first failed to notice another damning issue. The integration sites—the sequence of the human DNA either side of the virus—were *identical* to integration sites sequenced from experimentally infected cells in the same laboratory.^20,21^ The chances of this being coincidence is so vanishingly small as to not be worth bothering with. Alter had published a laboratory contamination (which he ultimately retracted^22^). Of course, to Mikovits and the activists supporting her claims, these were the increasingly desperate ravings of a “scientific establishment” trying to silence her.

At this point, the National Institute of Health and the U.S. blood transfusion service got involved. If there was a new retrovirus infecting untold number of Americans, they needed to know right now. Graham Simmons led a group from the blood transfusion service, who collaborated with Mikovits to try and reproduce the WPI's findings. At the same time, the National Institutes of Health tasked Ian Lipkin, a well-respected “virus hunter” from Columbia University, to set up a prospective reproduction study of ME/CFS patients to answer the question once and for all. Again, Mikovits and WPI agreed to collaborate.

There was disquiet brewing in Bob Silverman's team. They had sent Mikovits XMRV as a circular DNA form, known as a plasmid, which allows the viral genome to be grown up safely in bacteria. Included in the plasmid was a gene for resistance to the antibiotic, neomycin. Sometime later he had received CFS and healthy samples from the WPI to recheck, which he did according to Mikovits's protocol and confirmed her data: the CFS samples were almost all positive for XMRV, with only a 2 of 50 healthy controls positive. Now Silverman was worried. He pulled the samples from the WPI out of the freezer, but this time, instead of testing for XMRV, he did two other tests in case something more serious than a laboratory contamination had happened. He ran tests for the presence of both the neomycin resistance gene and the hybrid CMV promoter he had engineered into the virus when he cloned it.^23^ In all of the WPI samples that tested positive for XMRV, both could be detected, meaning that these human samples contained the plasmid he had sent to the WPI, furthermore, only in the ME/CFS samples. Not only were the original published results unsound, but also the most likely explanation was that the ME/CFS samples had been deliberately spiked with the plasmid—*prima facie* evidence of scientific fraud.

At about the same time, John Coffin, an expert on mouse endogenous retroviruses at Tufts University, Boston, and his colleagues Vinay Pathak and Wei Shu Hu who ran laboratories at the National Cancer Institute in Frederick, MD, were puzzling over the origins of XMRV. Coffin's group had found parts of the XMRV genome in strains of laboratory mice.^24,25^ Not the entire virus but certainly a plausible source for contaminating diagnostic tests if even a tiny amount of mouse DNA was around in the laboratory.^10,11^ Pathak and Hu had meanwhile been trying to trace the origin of the human prostate cancer cell line 22RV1. Delving through the records, they found that 22RV1 had been established at the Cleveland Cancer Center in the 1990s using a fairly common trick. To get difficult cells to grow from human cancers, parts of the tumor were injected under the skin of laboratory mice lacking an immune system. Once the tumor grew, it would be excised and passed on to a new mouse until a cell line that grew well in culture was established. Not only that, in the freezers of the laboratories where the cell line was derived, they found stored samples of each of these passages. Working backward through the samples, they found that the human prostate tumor from which 22RV1 had been derived was not originally XMRV positive, but the virus had appeared during the sequential grafting of the cells in mice.^26^ Not only that, the mouse tissue in the sample revealed that the mouse strain used harbored intact endogenous mouse retroviruses identical in parts to XMRV. What had clearly happened was that, by chance, two of these endogenous retroviruses had begun producing infectious viruses in the mouse bearing the tumor, they had infected the human cells and recombined together to make XMRV! The virus had never existed outside the laboratory that derived the cells and thus could not be causing human disease.

*Science* published both the negative blood service study that found no evidence of XMRV in a new batch of ME/CFS patients and Coffin, Hu and Pathak's study documenting the natural history of XMRV.^26,27^ Still Mikovits and the WPI were unmoved, and some of the activists became even more abusive to the scientists trying to sort this mess out. Then Silverman's group wrote to *Science* to explain that they had found evidence of plasmid DNA spiking in the WPI samples and, therefore, were not confident of the veracity of their part of Mikovits's article. Although they vociferously refuted any accusation of wrong doing, Mikovits and the WPI authors agreed to partially retract part of the original article,^23^ but maintained this was a technical problem and their new data would right these problems: a wholly unsatisfactory state of affairs. As Jonathan Stoye, a U.K. retrovirologist at what is now the The Francis Crick Institute, observed, the “paper was dying figure by figure; how long would this need to be dragged out?”

The end came swiftly.^28^ A retrovirology PhD student called Abbie Smith had been following the XMRV saga on her blog, ERV,^*^ with an ever-increasing skepticism of Mikovits and her work. Mikovits had presented a talk in which she expanded on the XMRV story, now saying that this was the tip of the iceberg and that it was likely that many other XMRV-like viruses were responsible for human conditions including autism (for which there has never been the slightest hint of evidence). In the presentation, Mikovits showed a slide purporting to show the expression of mouse retrovirus proteins in human blood cells when treated with a chemotherapy drug, 5-azacytidine (5-AZA). Despite her contention that this proved human infection by mouse retroviruses, this in fact is a well-known phenomenon. 5-AZA activates endogenous retroviruses common to humans and mice, and the antibodies against mouse retroviruses cross-react with these proteins. But there was something odd. One anonymous observer noticed a similarity between the slide and a figure in Mikovits's *Science* article. However, in the article, instead of the samples being labeled plus or minus 5-AZA treatment, they were labeled as ME/CFS patient or healthy control. By chance, Mikovits made the PowerPoint slides publically available. Upon uncropping the image, one could see the original handwriting on the figure showing indeed these were cells treated with 5-AZA, the implication being that it had been deliberately mislabeled to imply that the ME/CFS sample was infected with XMRV. The anonymous source sent the evidence to Smith who, in a gleefully theatrical blogpost, compared the slide with the article figure showing that they were identical. *Science* was aghast. The editor-in-chief Bruce Alberts took the highly unusual step of retracting the whole article without Mikovits or the WPI's agreement because it was now clear that it was fraudulent and scientifically invalid.

Mikovits was swiftly fired from the WPI who accused her of insubordination and misappropriating documents.^28^ After fleeing to California to avoid arrest, Mikovits returned documents to the WPI that a member of her laboratory had taken for her, and the charges were dropped. She was allowed to continue collaborating with Ian Lipkin and Frank Ruscetti, and they eventually published their “replication” study of the original WPI article showing that there was no evidence of human infection by XMRV in ME/CFS.^29^ And after that, we, as a field, heard little of her. In a final postscript to the whole sorry XMRV saga, the authors of the original study who identified XMRV in prostate cancer retracted their article^30^ on the basis that tumor samples reanalyzed found no evidence of XMRV and concluded that the presence of 22RV1 cells in the laboratory had contaminated the samples.^31^

Of course, in our time of social media and YouTube, conspiracy theories never die, they just re-emerge and become adapted to new hosts. Mikovits's statement that she thought XMRV might be the cause of autism has been picked up by the antivaccine movement led by the disgraced British ex-doctor, Andrew Wakefield, who himself had his medical license revoked for falsely linking the measles, mumps, and rubella vaccine to childhood developmental problems. The narrative that she was the victim of an establishment cover up to stop the world knowing what, if true, would have been the greatest scandal in medical history plays very well to those who cannot be persuaded by incontrovertible evidence that vaccines are safe and there is no evidence that they “trigger” autism. Mikovits has expanded her claims to state that XMRV and other mouse retroviruses in vaccines underlie a massive epidemic in chronic disease as well as autism, for which, of course, there is no evidence because it is “being suppressed.” She has further hitched her wagon to a growing movement of “COVID-denialists,” primarily composed of extreme right- and left-wing conspiracists who believe either the virus does not exist, is not responsible for the deaths being reported, or even was released as a means for shadowy forces (take your pick of the usual members) to control the world. She explicitly claims that a “new influenza virus vaccine” made in dog cells trialed for the past few years is riddled with coronaviruses and that is how SARS-CoV2 entered humans (forgetting that coronaviruses kill cells, and thus any such manufacture of a vaccine in infected cells, dog or otherwise, is impossible). Lastly, she claims to have accumulated this evidence over the years, but it has been suppressed by political forces led by Dr. Fauci. She claims he has had it in for her since she and her mentor Ruscetti isolated HIV-1 in the 1980s but were prevented by Fauci from publishing because another NIH laboratory led by Bob Gallo (who had discovered two previous human retroviruses) was already on the case. There is not a shred of evidence for this claim, and Mikovits herself only joined Ruscetti's laboratory after HIV-1 had been discovered by a French group in Paris. The discovery of HIV indeed led to a great political row between the United States and the French about who could claim credit, as detailed in the movie *And the Band Played On*, but whatever the rights or wrongs, Judy Mikovits played no role.

So, all in all Mikovits has form as a serial scientific fantasist who has consistently made unsubstantiated claims about mouse retroviruses as the cause of a number of human diseases. The only “evidence” ever published by her was unequivocally shown to stem from laboratory contamination and explicit fabrication of data. She was never a leading researcher in the field; her doctoral studies were only very minor contributions to the field and until the XMRV debacle, very few had ever heard of her. Her subsequent “research” took advantage of people desperate to have an explanation for their debilitating symptoms, giving them false hope and many a false narrative where they are the victims of a massive medical cover up. In doing so, she also trashed the reputation of her former mentor, Frank Ruscetti. Her reappearance now as an apparently maligned “scientific leader” challenging the orthodoxy on vaccines and COVID-19 would be a source of eye-rolling were she not being taken seriously by countless Internet warriors posting and reposting the trailer for Plandemic. Her claims have been picked up by right-wing commentators in the United States desperate to show that the lockdown measures taken against COVID-19 are a pernicious over-reaction designed to damage President Trump, whose inconsistent handling of the crisis has garnered a huge amount of criticism. In doing so, she is playing with fire in the heightened atmosphere of our “fake news” era. It is incumbent on scientists to call this out for what it is: fabricated nonsense. There is no legitimate debate to be had on these issues, and any credence given to these dangerous conspiracies will lead to even greater suffering resulting from COVID-19. Steer well clear of Plandemic and the claims of Judy Mikovits.

## Notes

^*^ https://scienceblogs.com/erv
